# PM_2.5_ pollution in Texas: a geospatial analysis of health impact functions

**DOI:** 10.3389/fpubh.2023.1286755

**Published:** 2023-12-01

**Authors:** Luke Bryan, Philip Landrigan

**Affiliations:** ^1^Boston College, Chestnut Hill, MA, United States; ^2^Centre Scientifique de Monaco, Monaco, Monaco

**Keywords:** air pollution, particulate matter, PM_2.5_, Texas, county, census tracts, health impact functions

## Abstract

**Background:**

Air pollution is the greatest environmental threat to human health in the world today and is responsible for an estimated 7–9 million deaths annually. One of the most damaging air pollutants is PM_2.5_ pollution, fine airborne particulate matter under 2.5 microns in diameter. Exposure to PM_2.5_ pollution can cause premature death, heart disease, lung cancer, stroke, diabetes, asthma, low birthweight, and IQ loss. To avoid these adverse health effects, the WHO recommends that PM_2.5_ levels not exceed 5 μg/m^3^.

**Methods:**

This study estimates the negative health impacts of PM_2.5_ pollution in Texas in 2016. Local exposure estimates were calculated at the census tract level using the EPA’s BenMAP-CE software. In BenMAP, a variety of exposure-response functions combine air pollution exposure data with population data and county-level disease and death data to estimate the number of health effects attributable to PM_2.5_ pollution for each census tract. The health effects investigated were mortality, low birthweight, stroke, new onset asthma, new onset Alzheimer’s, and non-fatal lung cancer.

**Findings:**

This study found that approximately 26.7 million (98.9%) of the 27.0 million people living in Texas in 2016 resided in areas where PM_2.5_ concentrations were above the WHO recommendation of 5 μg/m^3^, and that 2.6 million people (9.8%) lived in areas where the average PM_2.5_ concentration exceeded 10 μg/m^3^. This study estimates that there were 8,405 (confidence interval [CI], 5,674–11,033) premature deaths due to PM_2.5_ pollution in Texas in 2016, comprising 4.3% of all deaths. Statewide increases in air-pollution-related morbidity and mortality were seen for stroke (2,209 – CI: [576, 3,776]), low birthweight (2,841 – CI: [1,696, 3,925]), non-fatal lung cancers (636 – CI: [219, 980]), new onset Alzheimer’s disease (24,575 – CI: [20,800, 27,540]), and new onset asthma (7,823 – CI: [7,557, 8,079]).

**Conclusion:**

This study found that air pollution poses significant risks to the health of Texans, despite the fact that pollution levels across most of the state comply with the EPA standard for PM_2.5_ pollution of 12 μg/m^3^. Improving air quality in Texas could save thousands of lives from disease, disability, and premature death.

## 1 Background

Air pollution is the greatest environmental threat to human health in the world today and is responsible for an estimated 7–9 million deaths annually, according to the World Health Organization (1). In the United States, approximately 200,000 deaths are due to air pollution each year (2).

One of the most damaging air pollutants is PM_2.5_ (3), fine, invisible airborne particulate matter less than 2.5 micrometers in diameter (4). Most PM_2.5_ is formed by the incomplete combustion of fossil fuels - coal, gas, and oil - or biomass fuels such as wood (5). Other sources include wildfires, road dust, construction sites, landfills, industrial sources, and pollen (5–7). Due to their minuscule size, these tiny particles can enter deep into the lungs and in some cases enter the bloodstream (8, 9). PM_2.5_ pollution has been shown to damage the heart, lungs, and other organs and pose a significant risk to human health (8–12).

Exposure to PM_2.5_ can cause premature death (13–16) from ischemic heart disease, lung cancer, COPD and stroke (10, 16, 17). Exposure to PM_2.5_ also increases non-fatal incidence of these diseases as well as of diabetes and asthma (8, 10, 14, 15, 17–22). PM_2.5_ exposure may also cause pregnancy-related effects such as low birthweight, preterm birth, and stillbirth (9, 19, 23, 24). Recent studies have shown links between PM_2.5_ and neurocognitive disorders such as Alzheimer’s disease and IQ loss (17, 18, 25–27).

Recent studies show that PM_2.5_ exposure levels previously thought to be safe cause disease, disability, and premature death (1, 16). In light of these studies, the WHO lowered their recommended guideline for PM_2.5_ pollution to 5 μg/m^3^ in 2021 from their previous recommendation of 10 μg/m^3^ (16, 28). The United States EPA air quality standard for PM_2.5_ is 12 μg/m^3^, calculated as an annual mean (29).

Air pollution is widespread across the state of Texas – a large state in the southern United States with over 27 million people (30). A 2013 study examined data from 18 monitoring stations across Texas and found that the annual mean PM_2.5_ concentrations at all 18 sites were between 6 and 12 μg/m^3^ (31). While the study recognized that these values were below the EPA’s standard recommendation of 12 μg/m^3^, the PM_2.5_ levels at each of these monitoring stations were above 5 μg/m^3^. A separate 2022 study found similar results along the Texas-Mexico border, with all monitors observing PM_2.5_ concentrations greater than 5 μg/m^3^ across the year (32).

As previous studies have found hazardous levels of PM_2.5_ throughout the state of Texas, it is important to understand the impact of this pollution. This study seeks to provide localized estimates for health effects attributable to PM_2.5_ pollution. This type of exposomal analysis can provide insight into the burden of disease of air pollution, as PM_2.5_ not only causes premature death, but also disease and disability at all stages of life. This study performs a localized analysis so these costs can be assessed at the state, county, and census tract levels.

## 2 Methods

### 2.1 Overview

This study estimates the negative health impacts of PM_2.5_ pollution across the state of Texas using known health impact functions, local population data, observed health outcomes, and PM_2.5_ data. Population data were obtained from the US Census and were calculated at the census tract level (30). PM_2.5_ estimates came from the NASA 2016 daily PM_2.5_ dataset and were also estimated by census tract (33). Birth and death data, calculated at the county level, came from the Texas Department of State Health Services (34, 35). Lung cancer and asthma data came from the Texas.gov website (36, 37). Stroke data came from a 2011–2019 multi-year analysis of stroke prevalence in Texas (38). Data for Alzheimer’s disease incidence came from a 2023 national historical report from the Alzheimer’s Association (39). Health impact functions were selected for relevance to health outcomes of interest, their sample sizes, and by the quantity and quality of their citations in other studies. All non-vital health data were approximated at the state level. All estimates were made for 2016, because that is the most recent year for which information from the NASA daily PM_2.5_ dataset was available.

First PM2.5 estimates were generated for each census tract. These data were then joined to population data – also at the census tract level – using the EPA’s BenMAP-CE software. Then, all health impact functions were categorized by health outcome and input into BenMAP-CE. Census tract level calculations ran for each health impact function to estimate the number of health effects attributable to PM_2.5_ pollution. Results were then aggregated to observe county and state level trends.

### 2.2 PM_2.5_ exposure data

The particulate matter data used in this study came from a NASA-sponsored study on national PM pollution which used machine learning to generate daily PM_2.5_ estimates at millions of locations across the United States (33). For the purposes of this study, the 2016 annual means from 1.2 million sites were used.

To estimate air pollution levels across Texas, census tracts were geospatially mapped and compared to the coordinates of the PM2.5 estimates. The PM_2.5_ estimates – which are spaced approximately 1 km apart - overlapped with 5,189 of 5,265 (98.6%) census tracts. An average PM_2.5_ estimate was assigned to each of these tracts using all contained point estimates. For the 76 tracts with no PM_2.5_ intersections, the nearest PM_2.5_ estimate was determined, and that single value was treated as the tract average.

### 2.3 Population data

The population data used in this study are from the US Census website. The population dataset used was from 2016 and age-stratified. Age estimates were given in percentages of the total population, so exact figures for age were determined prior to any other calculations. Age was the only demographic factored into this study, as the exposure-response functions used did not vary on other demographic data.

### 2.4 Health effects data

Eight health outcomes were investigated in this study: all-cause mortality, ischemic heart disease mortality, lung-tracheal-bronchial cancer mortality, non-fatal lung cancers, strokes, new onset asthma, new onset Alzheimer’s, and low birth weight babies. These health effects were selected based on access to previous research and the ability to obtain incidence rate data. All datasets were applicable to 2016.

Data for all-cause mortality, ischemic heart disease mortality, lung-tracheal-bronchial cancer mortality, and low birth weight babies were obtained from the Texas Department of State Health Services (34, 36). Death counts were compared to the 2016 census population data to generate population-weighted incidence rates, while cases of low birthweight were compared to the total number of births. As all data were available at the county level, disease incidence rates were calculated by county.

The other health outcomes came from a variety of sources. Non-fatal lung cancer data were based on statewide incidence rates from 2015 to 2019 (37). Stroke data were based on 2016 prevalence in a multi-year analysis (38). New onset Alzheimer’s data were based on national records of age-based Alzheimer’s incidence (39). Asthma incidence was calculated from the statewide prevalence of childhood asthma in Texas (36). Data for these health outcomes were not available at the county level and were assumed to be constant throughout the state.

Studies for each of these health outcomes were identified as sources for health impact functions. The functions used and sources are listed in Table 1.

**Table 1:** Studies and references used for exposure-response functions.

Study Author	Health outcome	Year	Beta	Standard deviation	Ages
Krewski et al. (16)	Mortality, all cause	2009	0.0058268	0.0009628	30–99
Krewski et al. (16)	Mortality, ischemic heart disease	2009	0.021511	0.0020584	30–99
Krewski et al. (16)	Mortality, lung tracheal and bronchial cancer	2009	0.013103	0.0037945	30–99
Kloog et al. (21)	Stroke	2012	0.00343	0.00127	65–99
Ghosh et al. (23)	Low birthweight	2021	0.01094	0.00227	0–0
Gharibvand et al. (22)	Non-fatal lung cancer	2017	0.03784	0.01312	30–99
Kioumourtzoglou et al. (26)	New Onset Alzheimer’s	2016	0.13976	0.01775	65–99
Tetreault et al. (20)	New onset asthma	2016	0.044	0.0009	0–17

### 2.5 Statistical analyses

In generating the estimated exposure-response relationships, this study always assumed a log-linear model. This model factored population and incidence data with interpolated logarithmic measures of PM_2.5_ exposure to generate health-impact estimates for each census tract. This estimated the number of excess health outcomes due to PM_2.5_ air pollution based on previously calculated Beta coefficients. The formula for the log-linear model is below where Pop is the study population, BI is the baseline incidence, ΔPM is the annual particulate matter concentration in μg/m^3^, and β is the beta coefficient.


$$\Delta Y=\left(1-\frac{1}{e^{\beta *\Delta \mathrm{PM}}}\right)*\mathrm{BI}*\mathrm{Pop}$$

The EPA’s BenMAP-CE software was used to combine these datasets into health-impact estimations. BenMAP output an Excel file for each health-impact estimate. These files were treated as the results of the experiment.

## 3 Results

In 2016, the estimated total population of Texas was 26,956,435. Of this population, approximately 15,115,696 (56.1%) were 30 or older and 7,122,868 (26.4%) were below the age of 18. Estimated deaths and non-fatal lung cancers attributable to PM2.5 were examined for people 30–99 and estimated new onset asthma cases attributable to PM2.5 were examined for people 0–17. Thus, 22,238,564 people (82.5%) were included in this study’s at-risk population. Additionally, stroke and new onset Alzheimer’s cases attributable to PM2.5 were examined for people age 65–99. Approximately 3,096,174 people (11.4%) were above the age of 65. In 2016, there were 5,265 census tracts in Texas.

**Figure 1:**
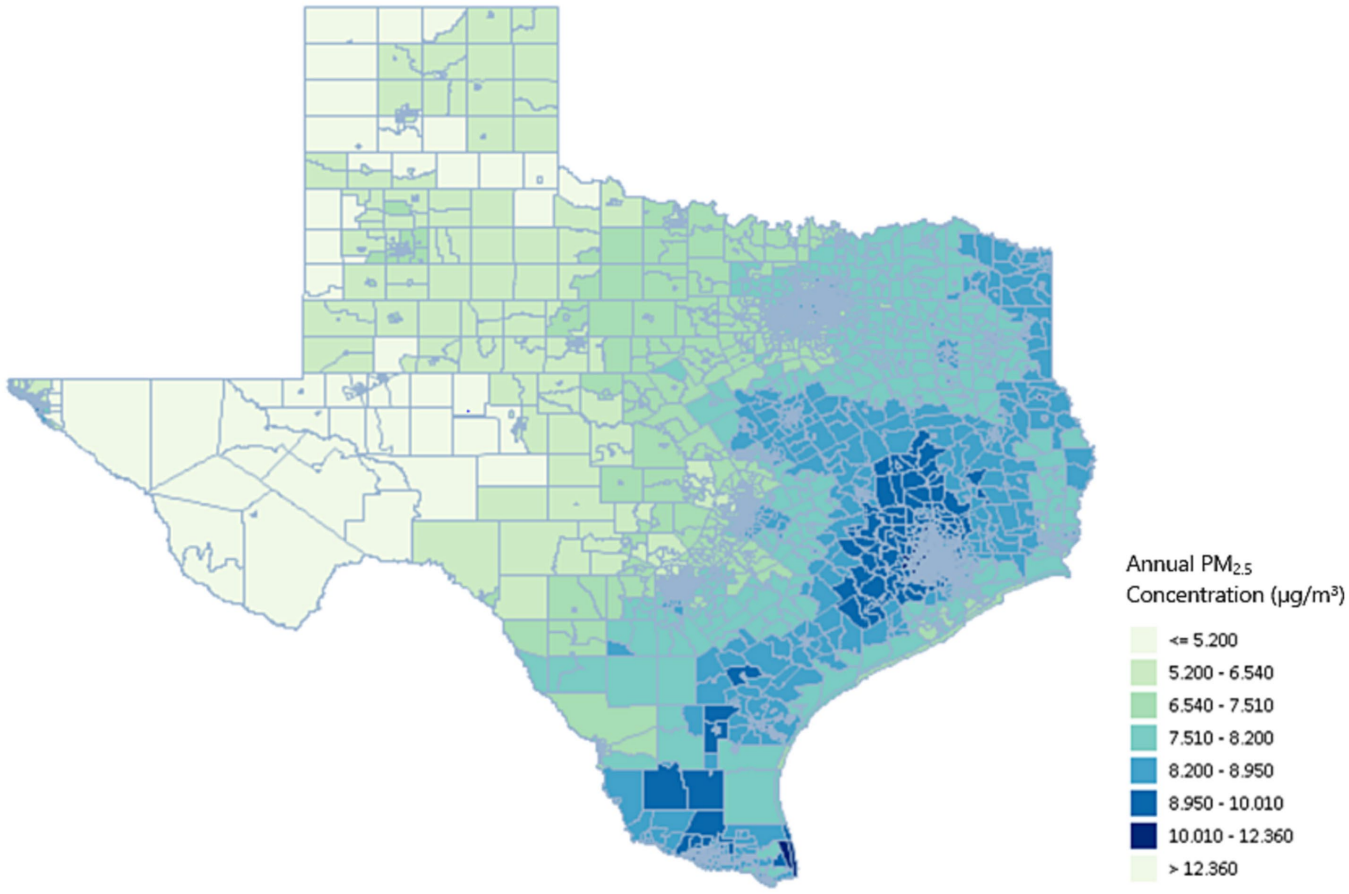
Texas PM_2.5_ concentrations (μg/m^3^) by census tract.

Air pollution estimates were created using daily PM_2.5_ pollution averages from 2016. Of the 5,265 census tracts, 5,227 had people in the at-risk population. The other 38 tracts contained airports, bodies of water, and other uninhabited or barely inhabited areas. This study estimated that of the 5,227 relevant census tracts, the minimum and maximum annual PM_2.5_ concentrations were 2.4 μg/m^3^ and 12.4 μg/m^3^, respectively. Of these tracts, 5,154 had PM_2.5_ levels that exceeded the WHO health recommendation of 5 μg/m^3^. These census tracts contained 98.9% of the population (26,664,944 people). 2,640,478 people (9.8%) resided in one of the 452 tracts that had annual PM_2.5_ levels greater than 10 μg/m^3^, and 19,053 (0.07%) resided in one of the four census tracts that exceeded the EPA standard of 12 μg/m^3^. The eastern part of the state had some of the highest air pollution levels, particularly around the Houston metropolitan area. The western parts of the state, which are generally less populated, contained most of the low-pollution census tracts. Figure 1 shows a tract-by-tract map of all estimated PM_2.5_ levels.

This study estimates that there were 8,405 (5,674, 11,033) premature deaths due to PM_2.5_ air pollution in Texas in 2016. Of the causes investigated, ischemic heart disease had the largest pollution-related incidence rate. There were an estimated 3,657 (3,009, 4,273) deaths due to ischemic heart disease and an estimated 755 (329, 1,152) deaths due to lung cancer attributable to air pollution.

Additional statewide estimates were generated for stroke (2,209 – CI: [576, 3,776]), low birthweight (2,841 – CI: [1,696, 3,925]), non-fatal lung cancers (636 – CI: [219, 980]), new onset Alzheimer’s* (24,575 – CI: [20,800, 27,540]), and new onset asthma (7,823 – CI: [7,557, 8,079]). Since these estimates were statewide, they were able to assess overall trends with PM2.5 data, but cannot measure local hotspots of disease. All statewide data for all health effects are listed in Table 2.

**Table 2:** Statewide estimates for health effects attributable to PM_2.5_.

Health Effect Attributable to PM_2.5_	Beta coefficient	Age range	Statewide estimate	Statewide confidence interval
Mortality, all cause	0.0058268	30–99	8,405	(5,674, 11,033)
Mortality, ischemic heart disease	0.021511	30–99	3,657	(3,009, 4,273)
Mortality, lung tracheal and bronchial cancer	0.013103	30–99	755	(329, 1,152)
Stroke	0.00343	65–99	2,209	(576, 3,776)
Low birthweight	0.01094	0–0	2,841	(1,696, 3,925)
Non-fatal lung cancers	0.03784	30–99	636	(219, 980)
New onset Alzheimer’s^a^	0.13976	65–99	24,575	(20,800, 27,540)
New onset asthma	0.044	0–17	7,823	(7,557, 8,079)

a Based on national incidence rate data.

Data for death and low birthweight were estimated at the county level. In a county-by-county analysis, Harris County had the largest number of estimated premature deaths at 1,368 (925, 1794). This is expected, as Harris County has nearly double the population of the next largest county. Dallas (673 – CI: [450, 880]), Bexar (541 – CI: [360, 710]), and Tarrant (561 – CI: [380, 740]) counties all had estimates of over 500 deaths per county.

Harris County also had the largest number of estimated low birthweight babies attributable to PM_2.5_ (623 – CI: [370, 850]). Dallas (265 – CI: [160, 360]), Bexar (203 – CI: [120, 280]), and Tarrant (194 – CI: [120, 270]) counties were the next largest (Table 3).

**Table 3:** Top 10 county estimates for vital health effects attributable to PM_2.5_.

County	Mortality, all cause	Mortality, lung tracheal and bronchial cancer	Mortality, ischemic heart disease	Low birth weight
Harris County	1,370 (925 to 1,790)	144 (62.5 to 217)	629 (518 to 731)	623 (371 to 854)
Dallas County	674 (454 to 883)	68.5 (29.5 to 104)	298 (245 to 348)	265 (157 to 365)
Bexar County	562 (378 to 736)	43.4 (18.7 to 65.8)	242 (199 to 283)	203 (120 to 279)
Tarrant County	541 (364 to 709)	65.5 (28.2 to 99.3)	215 (176 to 251)	194 (115 to 268)
Travis County	244 (164 to 320)	24.0 (10.3 to 36.3)	91.7 (75.3 to 107)	109 (64.7 to 150)
El Paso County	227 (153 to 297)	14.9 (6.43 to 22.6)	93.2 (76.6 to 109)	91.9 (54.6 to 127)
Hidalgo County	209 (141 to 273)	14.6 (6.31 to 22.1)	132 (109 to 154)	128 (76.3 to 176)
Collin County	191 (129 to 250)	20.4 (8.82 to 30.9)	83.1 (68.3 to 96.9)	78.9 (46.9 to 109)
Montgomery County	190 (128 to 248)	26.2 (11.4 to 39.5)	71.1 (58.5 to 82.7)	54.2 (32.3 to 74.3)
Fort Bend County	163 (110 to 214)	14.8 (6.4 to 22.3)	62.4 (51.4 to 72.6)	82.9 (49.4 to 114)

BenMAP also provided estimates for non-vital statistics at the county level. For example, Harris County experienced an estimated 1,520 (1,470 to 1,570) new asthma cases, 122 (42.0 to 181) non-fatal lung cancers, 355 (92 to 603) strokes, and 3,470 (2,980 to 3,810) new Alzheimer’s cases attributable to PM_2.5_ in 2016. All county-by-county data for vital and non-vital health effects can be found in the Supplementary Table S1.

## 4 Discussion

The main finding of this study is that air pollution by fine airborne particulate matter (PM_2.5_) is a major cause of disease and premature death in the state of Texas, despite the fact that most PM_2.5_ levels are below the US EPA standard of 12 μg/m^3^. These findings indicate that improving air quality in Texas could save thousands of lives from disease, disability, and premature death.

We found that there were 8,405 (5,674, 11,033) premature deaths due to PM_2.5_ pollution in Texas in 2016, comprising 4.3% of all deaths in the state. Harris, Dallas, Tarrant, and Bexar counties had air-pollution-related death tolls of 500–1,400. Statewide increases in air-pollution-related morbidity and mortality were seen for stroke (2,209 – CI: [576, 3,776]), low birthweight (2,841 – CI: [1,696, 3,925]), non-fatal lung cancers (636 – CI: [219, 980]), new onset Alzheimer’s (24,575 – CI: [20,800, 27,540]), and new onset asthma (7,823 – CI: [7,557, 8,079]).

A second key finding is that nearly 99% of census tracts across Texas had average annual PM_2.5_ concentrations over 5 μg/m^3^, a level that is associated with multiple adverse health effects and that the World Health Organization has declared dangerous. The highest levels of air pollution were seen in Harris County, which contains Houston. Harris County is highly industrialized and by far the most heavily populated county in Texas. The next highest annual PM_2.5_ estimates were seen in Fort Bend County, Waller County, and Montgomery County respectively, all of which share long borders with Harris County. These findings demonstrate that air pollution can cross political boundaries from one county to another and therefore requires large-scale, regional solutions that encompass entire airsheds.

This study has several limitations. The first is in the exposure data. The NASA daily PM_2.5_ dataset that we used to calculate air pollution exposures in the census tracts of Texas is a very highly verified source. It is based on a machine-learning model trained on daily data from across the state and country and is arguably the best available dataset. However, there are large, remote portions of the state of Texas that lack PM_2.5_ monitoring stations, and there is a degree of uncertainty in the estimates for those regions.

A second limitation is that all datasets used in this study were from 2016, 7 years prior to the conduct of the present analysis.

A third limitation is that we had to rely on non-localized data sources for information on health outcomes other than low birthweight and death. Incidence rate data for non-fatal lung cancers, strokes, and new onset asthma, were calculated from state-wide statistics and assumed to be evenly distributed throughout the state. This significantly reduced our ability to identify local hotspots of disease given the uneven distribution of PM_2.5_ concentrations (2.4–12.4 μg/m^3^). The incidence data for Alzheimer’s disease came from a national study, as there were no state-wide sources to be found.

## 5 Conclusion

While air pollution levels in most Texas counties comply with the current EPA standard for PM_2.5_ of less than 12 μg/m^3^, air pollution is nonetheless responsible for significant disease and death across the state. This finding indicates that the EPA standard is not protective of human health and will need to be reduced.

## Data availability statement

The original contributions presented in the study are included in the article/Supplementary material, further inquiries can be directed to the corresponding author.

## Author contributions

LB: Conceptualization, Data curation, Formal analysis, Funding acquisition, Investigation, Methodology, Software, Visualization, Writing – original draft, Writing – review & editing. PL: Funding acquisition, Methodology, Resources, Supervision, Writing – original draft, Writing – review & editing.
